# CDR1as regulated by hnRNPM maintains stemness of periodontal ligament stem cells via miR‐7/KLF4

**DOI:** 10.1111/jcmm.16541

**Published:** 2021-04-09

**Authors:** Xiuge Gu, Xiaoyu Li, Ye Jin, Zijie Zhang, Mengying Li, Dongxu Liu, Fulan Wei

**Affiliations:** ^1^ Department of Orthodontics School and Hospital of Stomatology Shandong University & Shandong Provincial Key Laboratory of Oral Tissue Regeneration & Shandong Engineering Laboratory for Dental Materials and Oral Tissue Regeneration Jinan China

**Keywords:** circRNA CDR1as, hnRNPM, KLF4, miR‐7, periodontal ligament stem cell, stemness

## Abstract

CDR1as is a well‐identified circular RNA with regulatory roles in a variety of physiological processes. However, the effects of CDR1as on stemness of periodontal ligament stem cells (PDLSCs) and the underlying mechanisms remain unclear. In this study, we detect CDR1as in human PDLSCs, and subsequently demonstrate that CDR1as maintains PDLSC stemness. Knockdown of CDR1as decreases the expression levels of stemness‐related genes and impairs the cell's multi‐differentiation and cell migration abilities, while overexpression of CDR1as increases the expression levels of stemness‐related genes and enhances these abilities. Furthermore, our results indicate that the RNA‐binding protein hnRNPM directly interacts with CDR1as and regulates its expression in PDLSCs. In addition, we show that CDR1as promotes the expression of stemness‐related genes in PDLSCs by inhibiting miR‐7‐mediated suppression of KLF4 expression. Collectively, our results demonstrate that CDR1as participates in the molecular circuitry that regulates PDLSC stemness.

## 1 INTRODUCTION

Periodontal ligament stem cells (PDLSCs) are undifferentiated mesenchymal stem cells present in the periodontal ligament (PDL) that have the capacity to self‐renew and generate differentiated cells. Human PDLSCs have been successfully isolated in vitro, and their stem cell properties have been identified.^1^ They are considered the main candidate stem cells for developing periodontal tissue regeneration techniques.^2^ Recently, PDLSC‐based periodontal regeneration was successfully established in dogs, rats and sheep.^3^ Furthermore, PDLSCs were successfully used to treat periodontal intrabony defects in a randomized clinical trial.^4^ Our previous studies have shown that PDLSC sheets can regenerate PDL‐like tissue in nude mice and miniature pigs.^5^, ^6^

A key step in tissue regeneration is the self‐renewal of stem cells. The evaluation of cell's self‐renewal capacity is the most direct way to measure ‘stemness’ of stem cells.^7^ Studies have demonstrated that stemness is modulated by the microenvironment, which includes small molecules, biologics, biomaterials and/or mechanical forces.^8^ Besides these external factors, stemness has been demonstrated to be regulated by complex genetic networks, including bone morphogenetic protein (BMP), Janus kinase/signal transducers and activators of transcription, Wnt and Notch signalling.^9^, ^10^, ^11^, ^12^ The exploration of stemness regulatory genes might provide a foundation for a more detailed understanding of stem cell biology, but the stemness regulatory mechanisms of PDLSCs have not yet been fully elucidated.

Circular RNA (circRNA) is a type of endogenous, non‐coding RNA with a covalently closed‐loop structure.^13^ Thanks to their tissue‐specific and highly conserved expression characteristics, circRNAs are considered to be biomarkers, or targets for diagnosis and treatment of diseases.^14^ Recently, circRNAs have been shown to participate in the maintenance of pluripotency of human embryonic stem cells,^15^ self‐renewal of intestinal stem cells,^16^ differentiation of osteoblasts and osteoclasts,^17^, ^18^ and the primary stage of rat liver regeneration.^19^ Therefore, we have been suggested that circRNAs might regulate the pluripotency and differentiation of PDLSCs. Previously, we have demonstrated that circRNAs are widely involved in PDLSC osteogenic differentiation through high‐throughput sequencing, and eight differentially expressed circRNAs were selected for qRT‐PCR validation.^20^ Among them, the circRNA CDR1as, an antisense transcript of cerebellar degeneration‐associated protein 1 (CDR1), was highly expressed in PDLSCs and up‐regulated during PDLSC osteogenic differentiation.^20^ Acting as a miR‐7 ‘sponge’, CDR1as was associated with human diseases and reported to exert biological functions, including activation of stem cell differentiation.^21^ However, the effects and mechanisms of CDR1as on the stemness of PDLSCs remain unclear.

In this study, we comprehensively explore the functional roles of CDR1as and its regulatory effects on PDLSC stemness properties, including proliferation, migration, osteogenic and adipogenic differentiation, and the expression of pluripotency‐associated genes. Furthermore, we present findings indicating that the RNA‐binding protein (RBP) hnRNPM directly interacts with CDR1as and regulates its expression in PDLSCs. In addition, we demonstrate that CDR1as promotes the expression of stemness‐related genes in PDLSCs by inhibiting miR‐7‐mediated suppression of KLF4 expression.

## 2 MATERIALS AND METHODS

All protocols for handling of human tissues were approved by the Research Ethics Committee of Stomatology Hospital of Shandong University, China (GR201710). Informed consent was obtained from all donors.

### 2.1 Cell culture and identification

Healthy human third molars were collected from 18‐ to 20‐year‐old patients at the Department of Oral Maxillofacial Surgery in Stomatology Hospital of Shandong University. PDLSCs were derived from the PDL of extracted teeth using the explant culture method and cultured in normal medium, consisting of α‐MEM (HyClone, South Logan, UT, USA), 10% foetal calf serum (FBS) (Gibco BRL, Grand Island, NY, USA), 100 U/mL penicillin, and 100 μg/mL streptomycin (Invitrogen, Carlsbad, CA, USA) at 37°C in 5% carbon dioxide. Then, 1 × 10^3^ cells were seeded in a 60‐mm dish and cultured for 10 days to assess the colony forming efficiency through crystal violet staining (Solarbio, Beijing, China). PDLSCs were characterized by the detection of cell surface markers (CD44, CD90, CD34 and CD45) through flow cytometric analysis.

### 2.2 Osteogenic induction, ALP and Alizarin Red staining

For osteogenic differentiation, PDLSCs were cultured with osteogenic inductive medium supplemented with 10 nM dexamethasone, 10 mM β‐glycerophosphate and 50 mg/L vitamin C (Sigma‐Aldrich, St. Louis, MO, USA). After 7 days, PDLSCs were fixed with 70% alcohol, and ALP staining was performed as described previously.^20^ After 21 days, PDLSCs were fixed with 4% paraformaldehyde and stained with 2% Alizarin Red (pH = 4.2) (Sigma‐Aldrich). To determine the relative amount of mineralized matrix, 10% w/v cetylpyridinium chloride (CPC) (Sigma‐Aldrich) was added to the stained plates, and samples were quantified by spectrophotometric absorbance at 562 nm.

### 2.3 Adipogenic induction and Oil Red O staining

For adipogenic differentiation, PDLSCs were cultured in normal medium and adipogenic inductive medium supplemented with 1 μM dexamethasone, 10 mg/L insulin, 0.5 mM 3‐isobutyl‐1‐methylxanthine and 0.2 mM indomethacin (Sigma‐Aldrich). After 14 days, PDLSCs were fixed with 4% paraformaldehyde and stained with Oil Red O (Sigma‐Aldrich). To determine the relative amount of oil deposition, isopropyl alcohol was added to the stained plates, and samples were quantified by spectrophotometric absorbance at 510 nm.

### 2.4 Identification of CDR1as

Total RNA was extracted with TRIzol (Takara, Tokyo, Japan) and reverse transcribed to cDNA. PDLSC gDNA was extracted using a TIANamp Genomic DNA Kit (Tiangen Biotech, Beijing, China). Divergent and convergent primers were designed to specifically target the circular junction site and the linear region of CDR1as for PCR amplification of cDNA and gDNA (Table 1). These amplification products were separated by agarose gel electrophoresis. The sequence of the divergent primer amplification product using cDNA was verified by Sanger sequencing.

**TABLE 1:** Gene primers for qRT‐PCR

Gene	Forward primer	Reverse primer
CDR1as	TGACATTCAGGTCTTCCAGTGT	TTGACACAGGTGCCATCGGA
KLF4	GCGCTGCTCCCATCTTTCTC	GGGGAAGTCGCTTCATGTGG
SOX2	CGATGCCGACAAGAAAACTT	CAAACTTCCTGCAAAGCTCC
OCT4	CCGAAAGAGAAAGCGAACCAG	AGAACCACACTCGGACCACATC
Nanog	CAGCAGATGCAAGAACTCTCCA	CATTGCTATTCTTCGGCCAGT
hnRNPM	AGTATGGCTGGTGTGGTGGT	TTGCACAGCTTCAATGGCT
GAPDH	TCATGGGTGTGAACCATGAGAA	GGCATGGACTGTGGTCATGAG
U6	TGGAACGCTTCACGAATTTGCG	GGAACGATACAGAGAAGATTAGC

### 2.5 Cell transfection

For CDR1as knockdown, lentiviral constructs (sh‐CDR1as #1 and sh‐CDR1as #2) were generated based on circular regions of CDR1as. For CDR1as overexpression, lentiviral constructs (ov‐CDR1as) were generated based on the whole CDR1as sequence. The same lentiviral vectors, yet containing non‐specific RNA oligonucleotides, denoted as sh‐NC and ov‐NC, were used as a negative control. For KLF4 and hnRNPM knockdown, four siRNA oligonucleotides complementary to different regions of human KLF4 and hnRNPM were designed and synthesized by Oligobio (Beijing, China). For the negative control, PDLSCs were transfected with a non‐specific RNA oligonucleotide. The shRNA and siRNA sequences are presented in Table 2.

**TABLE 2:** The sequences of shRNAs and siRNAs

Gene	Sequence (5′‐3′)
sh‐NC	TTCTCCGAACGTGTCACGT
sh‐CDR1as#1	GCCATCGGAAACCCTGGATAT
sh‐CDR1as#2	ACCCTGGATATTGCAGACACT
si‐NC	UUCUCCGAACGUGUCACGU
si‐KLF4#1	GGGUAUAAAUUAUAUCCGUTT
si‐KLF4#2	GGACCUACUUACUCGCCUUTT
si‐KLF4#3	CGAUCAGAUGCAGCCGCAATT
si‐KLF4#4	GAUCAACAUUUAUGACCUATT
si‐hnRNPM#1	GCACAGTATTTGTAGCAAA
si‐hnRNPM#2	GGATGAACATGGGCAGGAT
si‐hnRNPM#3	GGAAGATGCTAAAGGACAA
si‐hnRNPM#4	CCATTTGACTGTTTGCATT

### 2.6 Cell proliferation assay

The proliferation of PDLSCs was tested by EdU (5‐ethynyl‐20‐deoxyuridine) assay using a Cell‐Light EdU DNA Cell Proliferation Kit (RiboBio, Shanghai, China) according to the manufacturer's instructions. Furthermore, cell proliferation was also tested by a CCK8 kit (Doindo, Japan). Approximately 2 × 10^3^ transfected cells were incubated in 100 μL in triplicate in 96‐well plates. At 24, 48 and 72 hour, the CCK8 reagent (10 μL) was added to each well, and cells were incubated at 37°C for 2 hour. The optical density at 450 nm was measured using a spectrophotometer.

### 2.7 Cell migration assay

The effects of CDR1as on human PDLSC migration were evaluated in transwell chambers (Corning, NY, USA). Transfected PDLSCs in α‐MEM containing 0.1% FBS were cultured in the upper chamber, while the lower chamber held α‐MEM containing 10% FBS. After being incubated for 20 hour, migrated cells were fixed in 4% paraformaldehyde, stained with 0.1% crystal violet, and counted in six randomly selected high‐power microscopic fields per filter by blind evaluation. Cell migration was also evaluated by wound healing assay. A sterile pipette tip was used to scratch the monolayer. After 24 hour, the scratch was photographed. ImageJ v1.51 software was used to measure and calculate the distance that the cells had migrated.

### 2.8 Quantitative real‐time PCR (qRT‐PCR)

The qRT‐PCR reaction was performed with SYBR® Premix Ex Taq™ (Takara) on a Roche LightCycler®480 sequence detection system (Roche, Basel, Switzerland) following the manufacturer's protocol. The primer sequences are listed in Table 1. The primer of miR‐7 (Set Catalog #606) was purchased from Takara. Data were analysed using the 2^−△△CT^ method, with GAPDH and U6 as internal standards.

### 2.9 Antibodies

The antibodies used for Western blotting were the following: anti‐GAPDH (1:2000; Proteintech; 10494‐1‐AP), anti‐SOX2 (1:2000; Proteintech; 11064‐1‐AP), anti‐OCT4 (1:500; Proteintech; 11263‐1‐AP), anti‐Nanog (1:1000; Proteintech; 14295‐1‐AP), anti‐ALP (1:1000; Proteintech; 11187‐1‐AP), anti‐Runx2 (1:1000; Abcam; ab23981), anti‐hnRNPM (1:1000; Abcam; ab177957), anti‐KLF4 (1:500; Proteintech; 11880‐1‐AP) and horseradish peroxidase‐labelled secondary antibody (1:2000; Proteintech; SA00001‐2).

### 2.10 RNA‐protein pull‐down

In the RNA pull‐down assay, PDLSCs were lysed in co‐IP buffer, and samples were incubated with biotinylated oligo‐DNA probes against putative binding sites in CDR1as. A biotinylated random oligo probe was used as a negative control. Streptavidin‐coated magnetic beads were added to each binding reaction to pull down CDR1as and RBPs. The beads were then washed, and binding proteins were analysed by mass spectrometry to identify CDR1as binding proteins.

### 2.11 Dual‐luciferase reporter assay

In the dual‐luciferase reporter assay, 40 ng of luciferase reporter plasmid was transfected into 293T cells together with 100 nM miR‐7 mimic using Lipofectamine 3000. After transfection for 24 hour, the Renilla and firefly luciferase activities were measured separately using the Dual‐Luciferase Reporter Assay System (Promega, Beijing, China) following the manufacturer's instructions. The light intensity from Renilla luciferase was normalized to that of firefly luciferase.

### 2.12 Statistical analysis

Quantitative data are expressed as mean ± standard deviation (SD). Statistical analyses were performed using a Student's *t* test with SPSS 17.0 software. *P* <.05 was considered statistically significant.

## 3 RESULTS

### 3.1 Identification of PDLSCs and CDR1as

**FIGURE 1:**
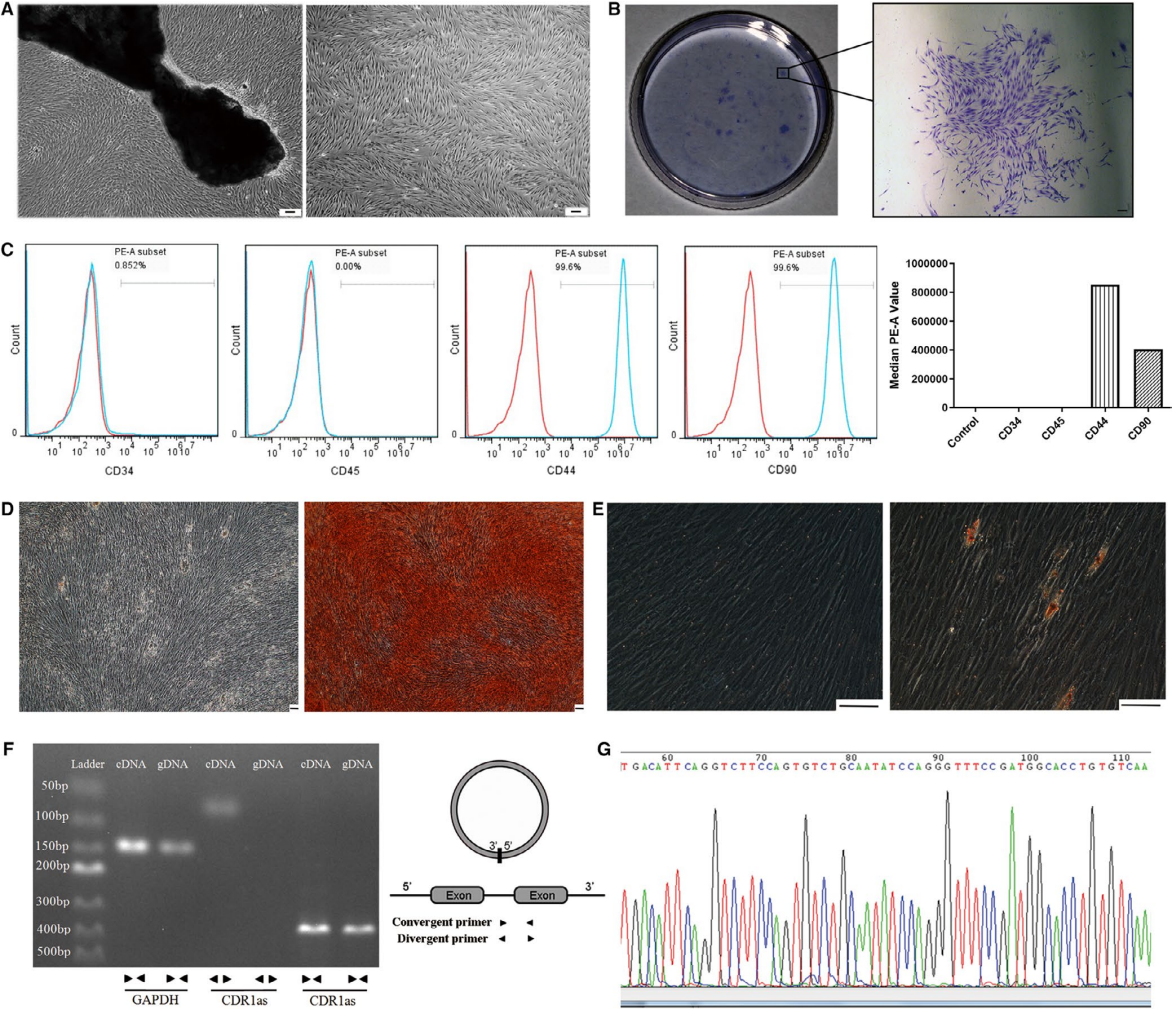
Identification and validation of PDLSCs and CDR1as. A. PDLSCs were derived from PDL explants on day 7 and cultured in normal medium until passage number 3. B. Single colonies formed after PDLSCs were cultured for 10 days. C. PDLSCs were characterized by detection of mesenchymal stem cell surface markers (CD44 and CD90) through flow cytometric analysis. Leukocyte markers (CD34 and CD45) were used as a negative control. D. Alizarin Red staining showing the mineralized matrix of osteo‐induced PDLSCs. E. Oil Red O staining showing the oil deposition in adipo‐induced PDLSCs F. Divergent and convergent primers of CDR1as were designed to specifically target the circular junction site and the linear region of CDR1as, respectively, for qRT‐PCR. The circular structure of CDR1as was validated by agarose gel electrophoresis. G. The head‐to‐tail splicing of the CDR1as RT‐PCR product was confirmed by Sanger sequencing. Scale bar (A, B, D, E), 100 μm

Primary cultured fibroblast‐like PDLSCs grew radially around the tissue explants and spread (Figure 1A). Subcultured PDLSCs were fairly uniform and exhibited strong proliferation capacity at passage number 3 (Figure 1A). The ability of PDLSCs to form cell clusters was shown by the formation of single colonies (Figure 1B). Cultured PDLSCs were positive for mesenchymal stem cell markers CD44 and CD90 and negative for leukocyte markers CD34 and CD45 (Figure 1C). After osteogenic induction, the amount of mineralized matrix was increased in PDLSC cultures (Figure 1D). Additionally, oil deposition was increased in adipo‐induced PDLSCs (Figure 1E). These results suggest that established PDLSCs maintained self‐renewal capacity and pluripotency.

The expression of CDR1as was validated by RT‐PCR followed by agarose gel electrophoresis. As a result, amplification products of convergent primers were detected using both cDNA and gDNA as template, while amplification products of divergent primers were only detected using cDNA (Figure 1F). The amplification product of divergent primers was Sanger sequenced (Figure 1G).

### 3.2 Knockdown of CDR1as impairs PDLSC stemness

**FIGURE 2:**
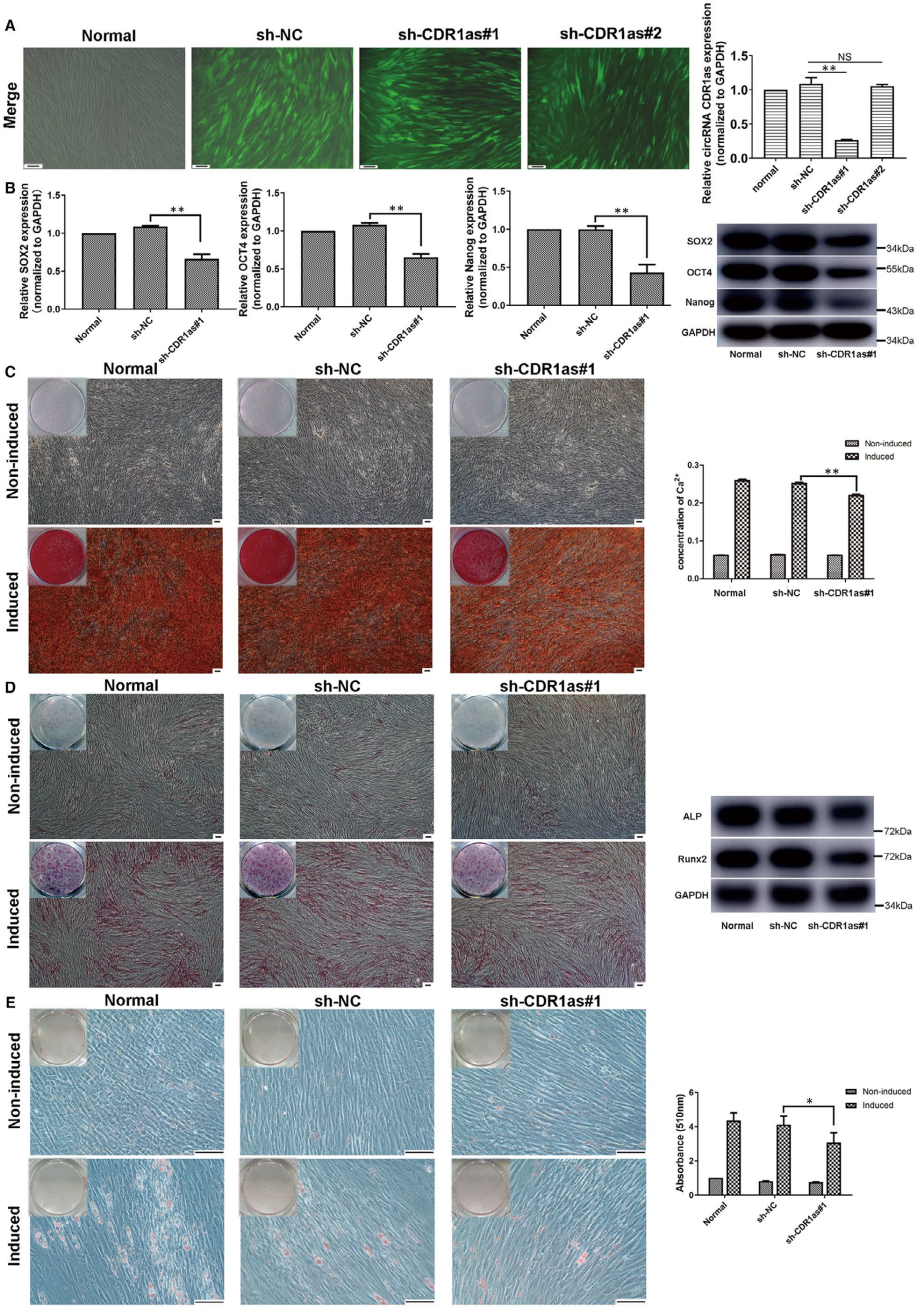
Knockdown of CDR1as down‐regulates the expression of stemness markers and PDLSC osteogenic differentiation. A. Approximately 80% of PDLSCs were green fluorescent, and thus successfully transfected by lentivirus, in the sh‐NC, sh‐CDR1as#1 and sh‐CDR1as#2 groups. The CDR1as expression levels in these groups were analysed by qRT‐PCR. The sh‐CDR1as#1 group was selected for subsequent experiments. B. mRNA and protein expression levels of stemness‐associated genes (SOX2, OCT4 and Nanog) as measured by qRT‐PCR and Western blot in the sh‐CDR1as#1 and sh‐NC groups. C. Mineralized matrix deposition by PDLSCs cultured with osteogenic inductive medium for 21 days, as demonstrated by Alizarin Red staining and quantified by CPC assay. D. Protein expression levels of ALP and Runx2 after culturing with osteogenic inductive medium for 7 days, as demonstrated by ALP staining and Western blot. E. Oil deposition of PDLSCs after culturing with adipogenic inductive medium for 21 days, as demonstrated by Oil Red O staining and quantified by optical absorbance at 510 nm after adding isopropyl alcohol. Scale bar (A, C, D, E), 100 μm. Quantitative qRT‐PCR data are presented as mean ± SD of three experiments. *P < .05; **P < .01; NS, not significant, by Student's *t* test

**FIGURE 3:**
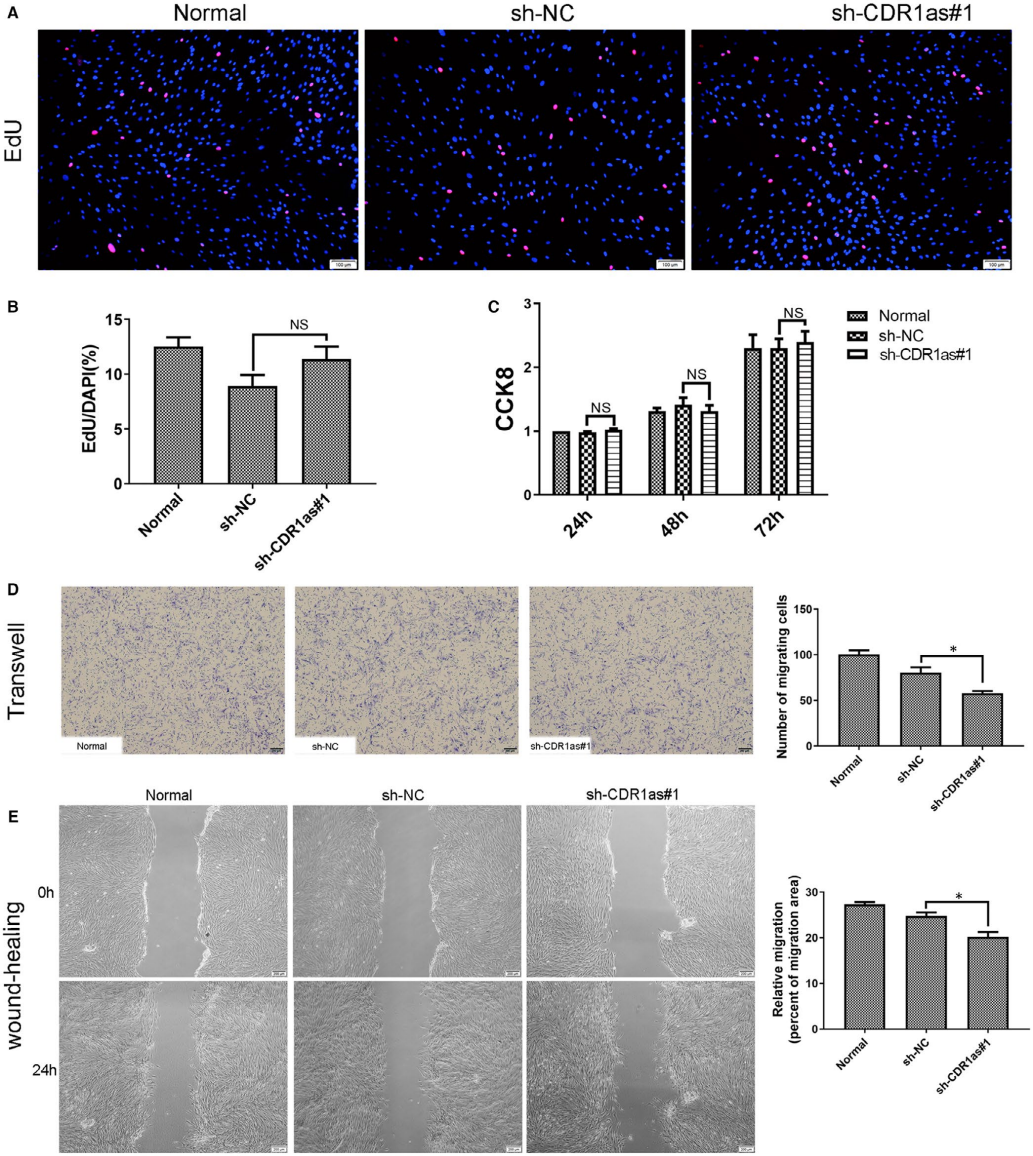
Knockdown of CDR1as inhibits migration and wound healing capacities of PDLSCs. A. DNA synthesis of PDLSCs was assessed by EdU assay after transfection with sh‐CDR1as#1 and sh‐NC for 48 h. B. Quantitative EdU assay data from A. C. Proliferation of PDLSCs in three groups was assessed at 24 h, 48 h and 72 h using a CCK8 kit. D. Migration ability of PDLSCs in three groups was assessed by transwell assay. Cells that migrated to the underside of the membrane were stained and counted. E. The average wound widths at 0 h and 24 h were analysed to assess the wound healing capacity of PDLSCs. Scale bar (A), 100 μm. Scale bar (D, E), 200 μm. Quantitative data are presented as mean ± SD. *P < .05; NS, not significant, by Student's *t* test

In the sh‐NC, sh‐CDR1as #1 and sh‐CDR1as #2 groups, approximately 80% of PDLSCs were green fluorescent, and thus successfully transfected (Figure 2A). Compared with the sh‐NC group, the expression of CDR1as in the sh‐CDR1as #1 group was reduced by approximately 80%. However, the expression of CDR1as in sh‐CDR1as #2 was not significantly reduced (Figure 2A). Therefore, sh‐CDR1as #1 was selected for subsequent experiments. The expression levels of stemness‐associated genes (SOX2, OCT4 and Nanog) were significantly down‐regulated in the sh‐CDR1as #1 group (Figure 2B). Next, we measured the multi‐differentiation, proliferation and migration abilities of PDLSCs. The amount of mineralized matrix in osteogenic‐induced PDLSC cultures was reduced in the sh‐CDR1as #1 group (Figure 2C). The expression of ALP and Runx2 were down‐regulated in the sh‐CDR1as #1 group (Figure 2D). Moreover, oil deposition in adipogenic‐induced PDLSCs was significantly decreased in the sh‐CDR1as #1 group (Figure 2E). The proliferation ability of PDLSCs was not significantly changed (Figure 3A−C). In addition, knockdown of CDR1as significantly impaired the migration ability of PDLSCs (Figure 3D and E). These results suggest that knockdown of CDR1as impairs PDLSC stemness.

### 3.3 Overexpression of CDR1as enhances PDLSC stemness

**FIGURE 4:**
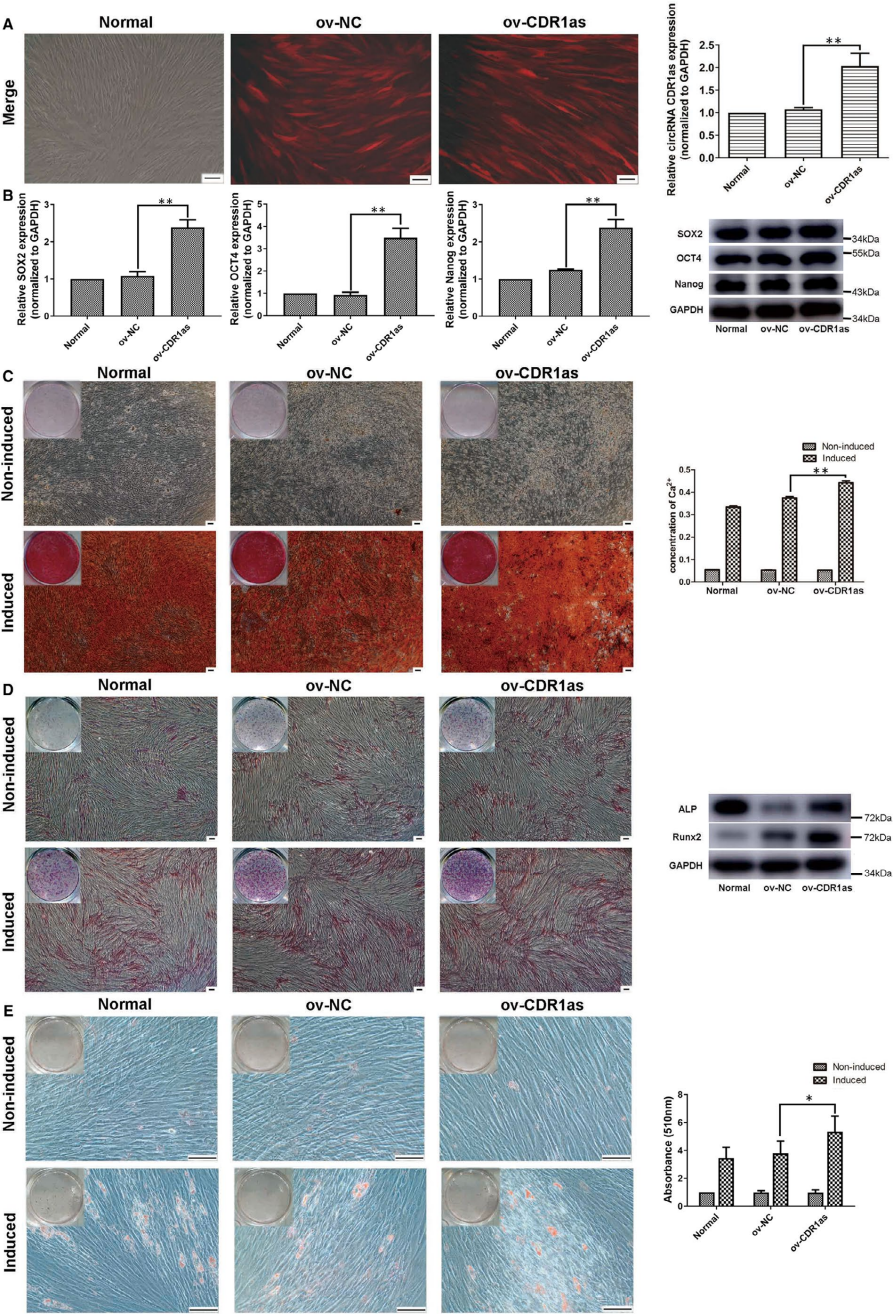
Overexpression of CDR1as up‐regulates the expression of stemness markers and PDLSC osteogenic differentiation. A. Approximately 80% of PDLSCs were red fluorescent, and thus successfully transfected by lentivirus, in the ov‐NC and ov‐CDR1as groups. The expression levels of CDR1as in these groups were analysed by qRT‐PCR. B. mRNA and protein expression levels of stemness‐associated genes (SOX2, OCT4 and Nanog) as measured by qRT‐PCR and Western blot in the ov‐CDR1as and ov‐NC groups. C. Mineralized matrix deposition by PDLSCs cultured with osteogenic inductive medium for 21 days, as demonstrated by Alizarin Red staining and quantified by CPC assay. D. Protein expression levels of ALP and Runx2 after culturing with osteogenic inductive medium for 7 days, as demonstrated by ALP staining and Western blot. E. Oil deposition of PDLSCs after culturing with adipogenic inductive medium for 21 days, as demonstrated by Oil Red O staining and quantified by optical absorbance at 510 nm after adding isopropyl alcohol. Scale bar (A, C, D, E), 100 μm. Quantitative qRT‐PCR data are presented as mean ± SD of three experiments. *P < .05; **P < .01; NS, not significant, by Student's *t* test

**FIGURE 5:**
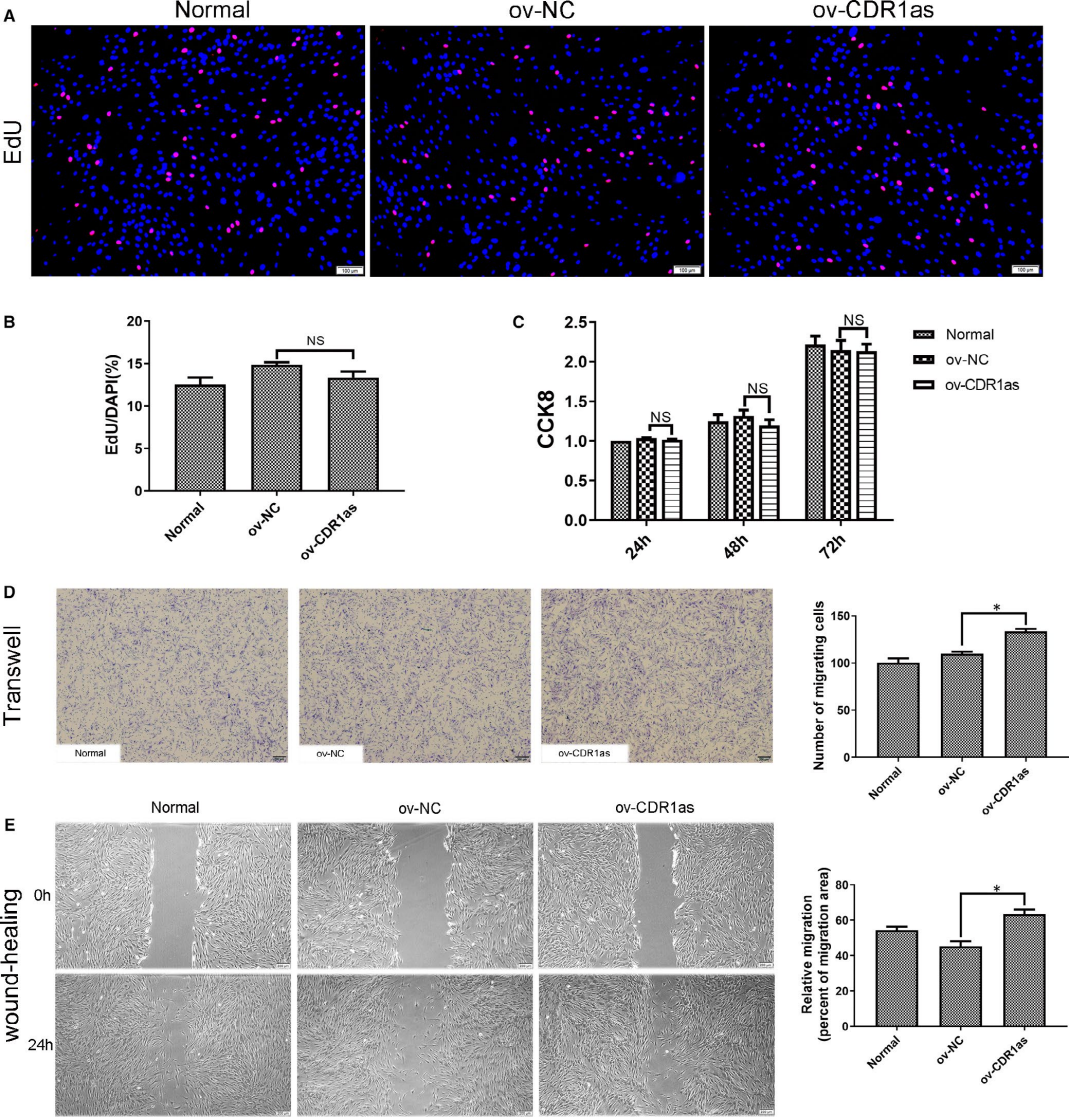
Overexpression of CDR1as maintains migration and wound healing capacities of PDLSCs. A. DNA synthesis of PDLSCs was assessed by EdU assay after transfection with ov‐NC and ov‐CDR1as#1 for 48 h. B. Quantitative EdU assay data from A. C. Proliferation of PDLSCs in three groups was assessed at 24 h, 48 h and 72 h using a CCK8 kit. D. Migration ability of PDLSCs in three groups was assessed by transwell assay. Cells that migrated to the underside of the membrane were stained and counted. E. The average wound widths at 0 h and 24 h were analysed using ImageJ 1.51 software to assess the wound healing capacity of PDLSCs. Scale bar (A), 100 μm. Scale bar (D, E), 200 μm. Quantitative data are presented as mean ± SD. *P < .05; **P < .01; NS, not significant, by Student's *t* test

Approximately 80% of PDLSCs were strongly red fluorescent, and thus successfully transfected (Figure 4A). The significant overexpression of CDR1as was demonstrated by qRT‐PCR (Figure 4A). The mRNA and protein expression levels of stemness‐associated genes (SOX2, OCT4 and Nanog) were significantly up‐regulated in the ov‐CDR1as group (Figure 4B). The amount of mineralized matrix in osteogenic‐induced PDLSC cultures was increased in the ov‐CDR1as group (Figure 4C). The expression of ALP and Runx2 was up‐regulated in the ov‐CDR1as group (Figure 4D). Oil deposition in adipogenic‐induced PDLSCs was significantly increased in the ov‐CDR1as group (Figure 4E). The proliferation ability of PDLSCs was not significantly changed (Figure 5A−C). In addition, overexpression of CDR1as significantly improved the migration ability of PDLSCs (Figure 5D and E). These results suggest that overexpression of CDR1as maintains PDLSC stemness.

### 3.4 The expression of CDR1as in PDLSCs is regulated by hnRNPM

**FIGURE 6:**
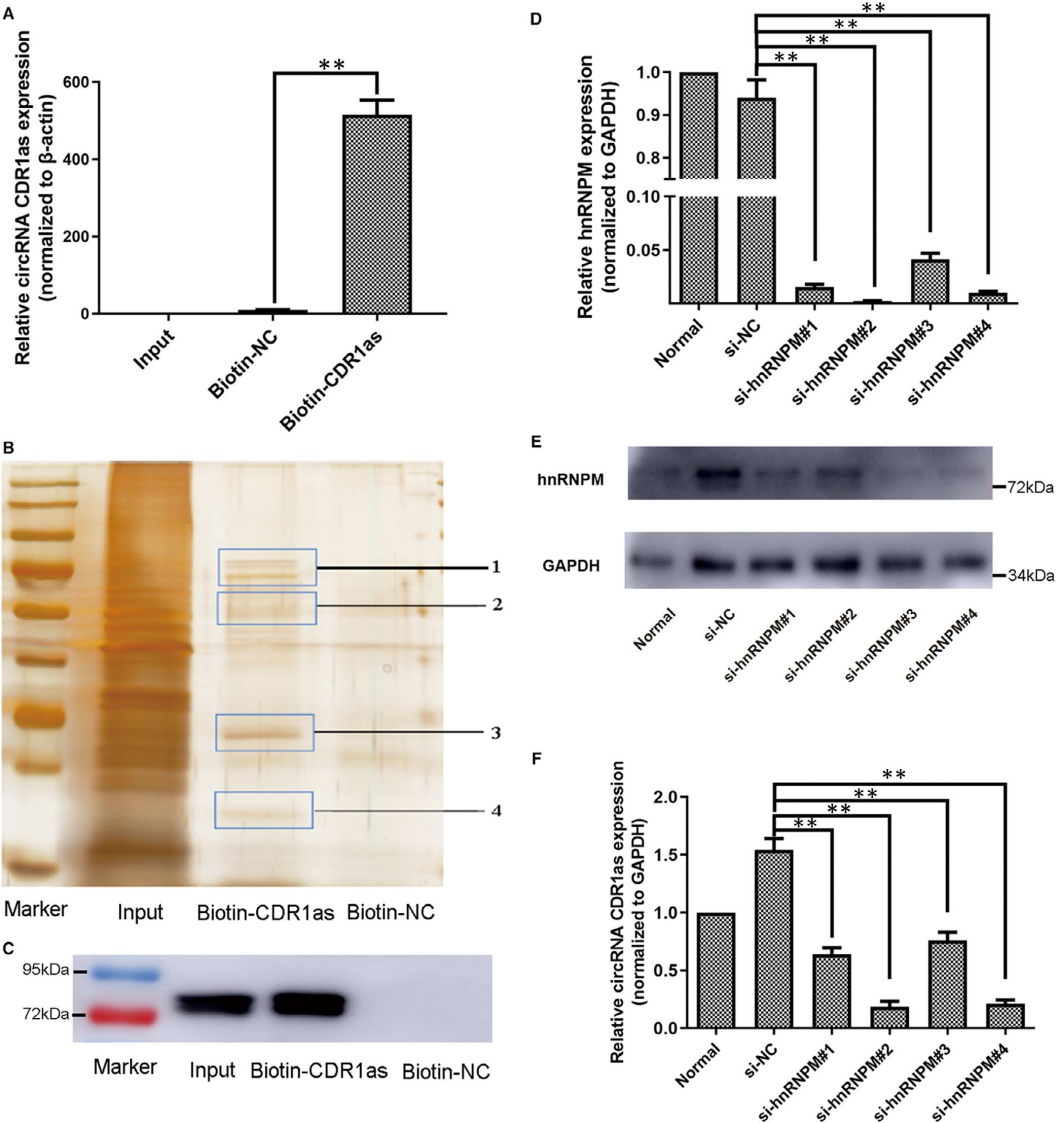
hnRNPM promotes the expression of CDR1as in PDLSCs. In the RNA pull‐down assay, the biotinylated CDR1as probe (biotin‐CDR1as) was designed to pull down CDR1as and RBPs. Biotinylated random oligo (biotin‐NC) was used as a negative control. A. Normalized CDR1as levels in input and biotin‐CDR1as and biotin‐NC elutions of the pull‐down assay, as measured by qRT‐PCR. B. Silver staining of eluted samples from the PDLSC pull‐down assay. C. hnRNPM protein levels in eluted samples from the pull‐down assay, as measured by Western blot. D. hnRNPM mRNA expression levels in PDLSCs transfected with si‐NC and four siRNAs targeting different regions of hnRNPM, as analysed by qRT‐PCR. E. hnRNPM protein expression levels in PDLSCs transfected with si‐NC and four siRNAs targeting different regions of hnRNPM, as analysed by Western blot. F. CDR1as levels in PDLSCs transfected with si‐NC and four siRNAs targeting different regions of hnRNPM, as analysed by qRT‐PCR. Quantitative qRT‐PCR data are presented as mean ± SD of three experiments. **P < .01, by Student's *t* test

As reported, RBPs might interact with circRNA and control its expression level.^22^, ^23^ To examine whether RBPs regulate the expression of CDR1as, we carried out an RNA pull‐down assay. The biotinylated CDR1as probe (biotin‐CDR1as) was designed to pull down CDR1as and RBPs. Biotinylated random oligo (biotin‐NC) was used as a negative control. CDR1as levels in the biotin‐CDR1as elution were much higher than in the biotin‐NC elution (Figure 6A). Next, we electrophoresed and silver stained the pull‐down products. Several bands were only found in the biotin‐CDR1as group (Figure 6B). After cutting out these bands, we identified 68 proteins that bind to CDR1as by mass spectrometry (Table S1). Among them, heterogeneous nuclear ribonucleoprotein M (hnRNPM) exhibited the highest protein abundance (emPAI). We also detected hnRNPM in the input and biotin‐CDR1as samples, but not in the biotin‐NC sample, which validates the binding of hnRNPM to CDR1as (Figure 6C). To determine whether hnRNPM is involved in the regulation of CDR1as expression, we designed four siRNA oligonucleotides to knockdown hnRNPM expression. The expression of hnRNPM was significantly lower in si‐hnRNPM groups #1‐4 than in the si‐NC group (Figure 6D and E). Moreover, our qRT‐PCR results show that the expression levels of CDR1as were significantly lower in si‐hnRNPM groups #1‐4 than in the si‐NC group (Figure 6F). Taken together, these results suggest that hnRNPM promotes the expression of CDR1as in PDLSCs.

### 3.5 CDR1as regulates stemness of PDLSCs via miR‐7 and KLF4

**FIGURE 7:**
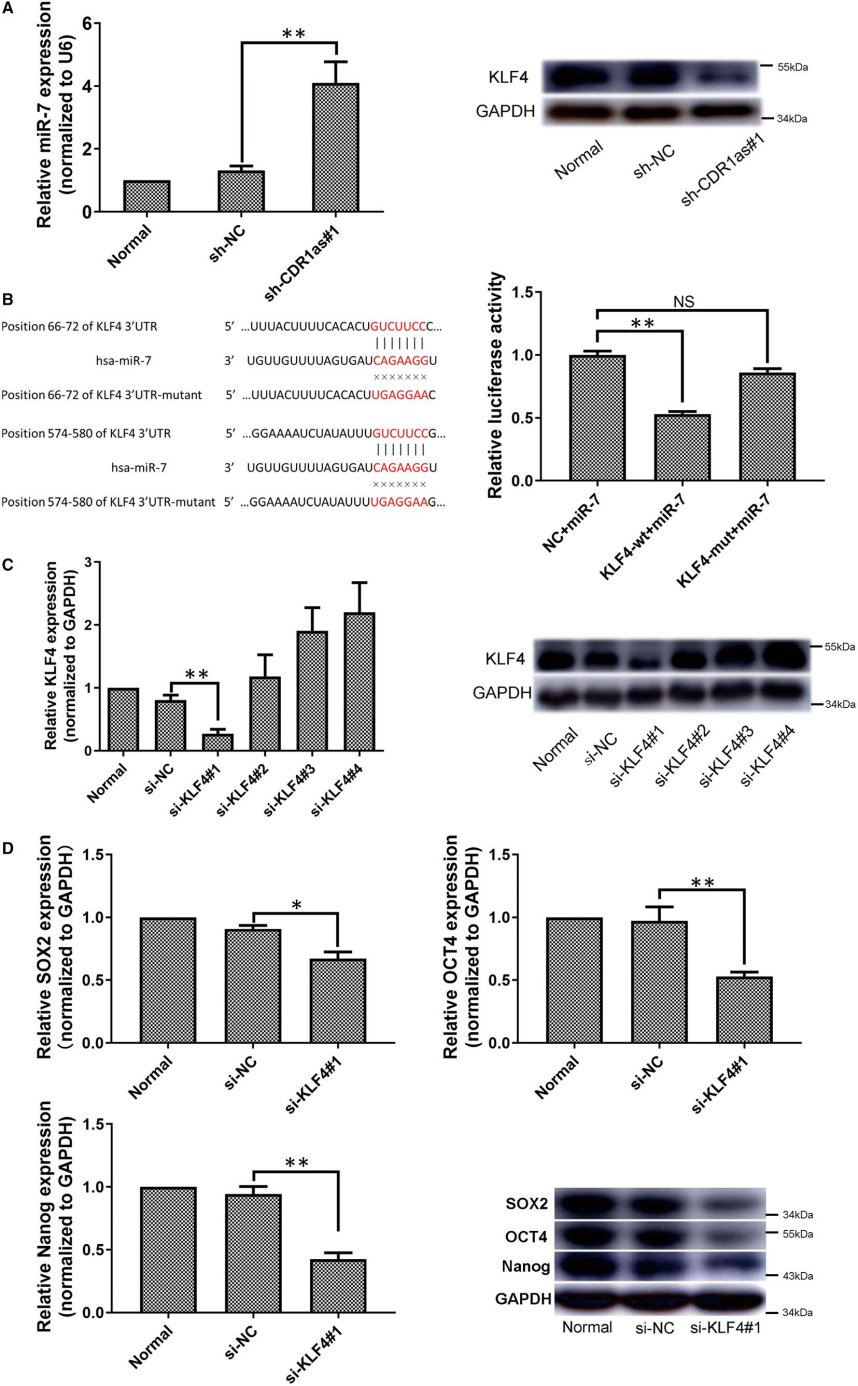
CDR1as regulates stemness of PDLSCs via miR‐7 and KLF4. A. The expression levels of miR‐7 and KLF4 in the sh‐CDR1as#1 and sh‐NC groups were analysed. B. Schematic illustration showing the differences between the two luciferase reporters, including one containing the complete KLF4 3′‐UTR sequence (KLF4‐wt) and one containing the KLF4 3′‐UTR sequence, with mutated sequences in the two miR‐7 binding sites (KLF4‐mut). The reporter assay showed the luciferase activity of KLF4‐wt and KLF4‐mut in 293T cells co‐transfected with miR‐7 mimics. C. KLF4 mRNA and protein expression levels in PDLSCs transfected with si‐NC and four siRNAs targeting different regions of KLF4, as analysed by qRT‐PCR and Western blot. D. mRNA and protein expression levels of stemness‐associated genes (SOX2, OCT4 and Nanog) in PDLSCs transfected with si‐NC and siRNA targeting KLF4, as measured by qRT‐PCR and Western blot. Quantitative qRT‐PCR data are presented as mean ± SD of three experiments. **P < .01, by Student's *t* test

It is known that CDR1as has approximately 70 conserved miR‐7 binding sites and performs biological functions by acting as a miR‐7 sponge.^18^ To determine whether CDR1as regulates the stemness of PDLSCs through interactions with miR‐7, we first showed by qRT‐PCR that the relative expression level of miR‐7 was up‐regulated in the sh‐CDR1as #1 group (Figure 7A). The expression levels of CDR1as and miR‐7 are negatively correlated. To further uncover the downstream molecules by which CDR1as and miR‐7 affect PDLSC stemness maintenance, we analysed potential target genes of miR‐7 using TargetScan7. Moreover, KLF4 expression was down‐regulated in the sh‐CDR1as #1 group (Figure 7A). Notably, we found that the 3′‐untranslated region (3′‐UTR) of the stemness‐related gene KLF4 contains two conserved miR‐7 binding sites (Figure 7B). Next, we validated the interaction of miR‐7 and KLF4 by dual‐luciferase reporter assay in 293T cells. Two luciferase reporters for KLF4 were constructed. The wild‐type (KLF4‐wt) reporter contained the complete 3’‐UTR sequence of KLF4, and the mutant‐type (KLF4‐mut) reporter contained the 3’‐UTR with mutated sequences in the two miR‐7 binding sites (Figure 7B). Our results indicate that overexpression of miR‐7 markedly decreases luciferase activity in the KLF4‐wt group, whereas this decrease was not observed in the KLF4‐mut group (Figure 7B). Collectively, these results suggest that CDR1as reduces the activity of miR‐7, up‐regulating KLF4 expression.

To verify whether KLF4 participates in PDLSC stemness regulation, four siRNA oligonucleotides complementary to different regions of human KLF4 were used to knockdown KLF4. Our results show that KLF4 expression was significantly lower in the si‐KLF4 #1 group than in the si‐NC group (Figure 7C). Furthermore, we examined the regulatory effects of KLF4 on PDLSC stemness. The mRNA and protein expression levels of stemness‐associated genes (SOX2, OCT4 and Nanog) were significantly down‐regulated in the si‐KLF4 #1 group compared with the si‐NC group (Figure 7D). Taken together, these results suggest that CDR1as maintains stemness of PDLSCs through direct interaction with miR‐7, up‐regulating KLF4 expression.

## 4 DISCUSSION

A critical requirement in periodontal tissue regeneration is the stemness of a PDLSC‐enriched population that retains the features of its original constituent cells.^24^ Stemness of PDLSCs is closely related to age, oestrogen levels, hypoxia and the inflammatory microenvironment, strongly limiting their usefulness in tissue engineering.^25^, ^26^, ^27^, ^28^ Maintaining stemness of PDLSCs might provide a new strategy for repair of injured periodontal tissue and in situ regeneration. With the ongoing development in bioinformatics, many non‐coding RNAs have been found to participate in stemness regulation.^29^ Recently, circRNA, another type of endogenous non‐coding RNA, has been demonstrated to control plasticity of mesenchymal stem cells.^30^ In our previous study, we indicated that CDR1as is highly expressed in PDLSCs and is up‐regulated during PDLSC osteogenic differentiation.^20^ In this study, we comprehensively explored the functional role of CDR1as in regulating PDLSC stemness properties.

Our study suggests that knockdown of CDR1as decreases the expression levels of stemness‐related markers (SOX2, OCT4 and Nanog), whereas overexpression of CDR1as increases their expression levels. SOX2, OCT4 and Nanog have been demonstrated to play crucial roles in the maintenance of stemness.^31^, ^32^ Previous studies have indicated that they are also expressed in PDLSCs and regulated by various factors and genes.^27^, ^33^, ^34^ Besides stemness‐related markers, pluripotency is another stemness property of stem cells. Differentiation potential of PDLSCs to cementoblasts, osteoblasts, fibroblasts during regenerative therapy is more critical. Our previous studies have also shown that PDLSC sheets differentiate to odontoblasts/cementoblast‐like cells and promote PDL‐like tissue regeneration in nude mice and miniature pigs.^5^, ^6^ Differentiation of mesenchymal stem cells is regulated by a complex network of transcription factors and signalling pathways.^35^ Our previous study indicated that a variety of circRNAs is differentially expressed upon PDLSC osteogenic differentiation and might play important regulatory roles.^20^ In this study, we suggest that CDR1as could enhance the osteogenic and adipogenic differentiation of PDLSCs.

Cell migration is another stemness property of stem cells. Studies have demonstrated that signalling molecules like substance P, stromal‐derived factor 1a and stem cell factor participate in the regulation of this process.^36^ These molecules activate endogenous mechanisms for self‐repair of injured tissue and in situ regeneration techniques. Our study suggests that CDR1as maintains the cell migration ability of PDLSCs and might therefore provide a new strategy for in situ periodontal tissue regeneration. Additionally, we found that the proliferation capacity of PDLSCs is not significantly changed upon CDR1as knockdown or overexpression. Although cell proliferation and differentiation mostly show a remarkable inverse relationship, the regulation of the proliferation‐differentiation decision is tissue specific.^37^ Some specific examples have been reported of coincident occurrence of proliferation and differentiation. For instance, the cell proliferation capacity was not significantly changed upon elevation of odontogenic differentiation ability in bone morphogenetic protein 2 gene‐transfected stem cells from human tooth apical papilla (SCAP).^38^ Similarly, hypoxia had no effect on SCAP proliferation, but it evoked the up‐regulation of genes specific for osteogenic differentiation, neuronal differentiation and angiogenesis.^39^ In summary, our study has explored the functional roles of CDR1as and its regulatory effects on PDLSC stemness properties, including proliferation, migration, differentiation and the expression of stemness‐associated genes. Besides, other features could also indicate stemness potentials of PDLSCs, like telomerase activity. A long‐term culture of mesenchymal stem cells can result in a loss of their primary phenotype with reduction of telomerase activity.^40^ In this study, PDLSCs were used at passage number 3 to maintain stemness. Telomerase activity of stem cells could be regulated by transcription factors. For instance, SOX2 could enhance multipotential and self‐renewal of neural progenitor cells by maintaining telomerase activity.^41^ However, the effects of CDR1as on telomerase activity and stemness of long‐term culture PDLSCs remain unknown, which will be considered in our future studies.

A previous study indicated that the expression of circRNA has cell type‐specific features.^42^ In our previous study, we identified the differential expression of CDR1as between normal and osteo‐differentiated PDLSCs.^20^ These results suggest that the expression of CDR1as might be regulated by specific elements. Recently, RBPs were demonstrated to interact with circRNAs and to control their expression levels.^22^, ^23^ Indeed, in the present study, we found that hnRNPM directly interacts with CDR1as, and that hnRNPM knockdown impairs CDR1as expression. HnRNPM is an RBP that forms complexes with heterogeneous nuclear RNA. It has been reported that hnRNPM is involved in carcinogenesis, spinal muscular atrophy and cell differentiation by associating with pre‐mRNAs in the nucleus and influencing pre‐mRNA processing.^43^, ^44^, ^45^, ^46^, ^47^ The findings of the present study indicate that hnRNPM regulates the expression of CDR1as in PDLSCs.

CircRNAs are known to perform biological functions by regulating miRNAs and their downstream target genes. It is known that CDR1as has approximately 70 conserved miR‐7 binding sites and could therefore act as a miR‐7 ‘sponge’.^48^, ^49^ Here, we uncover the regulatory roles of CDR1as on miR‐7 and its target genes. Our results indicate that CDR1as can reduce the activity of miR‐7, up‐regulating KLF4 expression in PDLSCs. Moreover, we found that the 3′‐UTR of the stemness‐related gene KLF4 contains two conserved miR‐7 binding sites, which was validated by dual‐luciferase reporter experiments. Previously, miR‐7 had been reported to regulate angiogenesis, metastasis and invasion by modulating KLF4.^50^, ^51^, ^52^ To the best of our knowledge, we are the first to study the regulatory roles of miR‐7 and KLF4 in PDLSCs. We have shown that KLF4 knockdown decreases the expression levels of SOX2, OCT4 and Nanog in PDLSCs. A previous study reported that forced expression of OCT4, SOX2, KLF4 and Nanog, or other combinations of reprogramming factors could reprogram somatic cells into induced pluripotent stem cells.^53^ Overexpression of KLF4 can indeed support self‐renewal and pluripotency of stem cells.^54^ Therefore, we conclude that CDR1as regulates stemness of PDLSCs via miR‐7 and KLF4.

In conclusion, our results demonstrate that CDR1as promotes the pluripotent state of PDLSCs by inhibiting miR‐7‐mediated suppression of KLF4 expression, and hnRNPM can promote the expression of CDR1as in PDLSCs. These findings might provide new genetic strategies for PDLSC‐based periodontal regenerative medicine.

## CONFLICT OF INTEREST

The authors declare no conflict of interest.

## AUTHOR CONTRIBUTION

**Xiuge Gu:** Data curation (lead); Formal analysis (lead); Investigation (lead); Methodology (lead); Project administration (lead); Supervision (lead); Writing‐original draft (lead); Writing‐review & editing (lead). **Xiaoyu Li:** Data curation (equal); Formal analysis (equal). **Ye Jin:** Data curation (equal); Formal analysis (equal). **Zijie Zhang:** Data curation (equal); Formal analysis (equal). **Mengying Li:** Data curation (equal); Formal analysis (equal). **Dongxu Liu:** Formal analysis (supporting); Supervision (supporting). **Fulan Wei:** Data curation (supporting); Formal analysis (supporting); Funding acquisition (lead); Investigation (supporting); Methodology (supporting); Project administration (supporting); Supervision (supporting); Validation (lead); Writing‐original draft (supporting); Writing‐review & editing (supporting).

## Supporting information

Table S1

## Data Availability

The data that support the findings of this study are available from the corresponding author upon reasonable request.
