# Supplementary material for: CDR1as regulated by hnRNPM maintains stemness of periodontal ligament stem cells via miR‐7/KLF4
Source: J Cell Mol Med. 2021 Apr 9;25(9):4501–15. doi: 10.1111/jcmm.16541 (PMC8093972; doi:10.1111/jcmm.16541)
Supplement: Supplementary file 1 — Table S1 [file JCMM-25-4501-s001.docx]

Table S1. The 68 proteins binding to CDR1as in the RNA-protein pull-down assay

| PF Number | Accession | Score | Mass | Matches | Sequences | emPAI | Protein description |
| --- | --- | --- | --- | --- | --- | --- | --- |
| 1.1 | P04264 | 3670 | 66170 | 140 (110) | 45 (38) | 11.43 | Keratin, type II cytoskeletal 1 OS=Homo sapiens OX=9606 GN=KRT1 PE=1 SV=6 |
| 1.2 | P35908 | 1591 | 65678 | 83 (67) | 33 (29) | 6.04 | Keratin, type II cytoskeletal 2 epidermal OS=Homo sapiens OX=9606 GN=KRT2 PE=1 SV=2 |
| 1.3 | P13647 | 674 | 62568 | 52 (36) | 33 (25) | 2.78 | Keratin, type II cytoskeletal 5 OS=Homo sapiens OX=9606 GN=KRT5 PE=1 SV=3 |
| 1.4 | P48668 | 587 | 60273 | 41 (30) | 23 (20) | 2.05 | Keratin, type II cytoskeletal 6C OS=Homo sapiens OX=9606 GN=KRT6C PE=1 SV=3 |
| 2.1 | P35527 | 2090 | 62255 | 103 (76) | 32 (26) | 5.05 | Keratin, type I cytoskeletal 9 OS=Homo sapiens OX=9606 GN=KRT9 PE=1 SV=3 |
| 2.2 | P13645 | 1929 | 59020 | 100 (71) | 34 (29) | 6.04 | Keratin, type I cytoskeletal 10 OS=Homo sapiens OX=9606 GN=KRT10 PE=1 SV=6 |
| 2.3 | P02533 | 623 | 51872 | 39 (31) | 24 (19) | 2.88 | Keratin, type I cytoskeletal 14 OS=Homo sapiens OX=9606 GN=KRT14 PE=1 SV=4 |
| **3** | **P52272** | **1369** | **77749** | **83 (61)** | **40 (33)** | **6.59** | **Heterogeneous nuclear ribonucleoprotein M OS=Homo sapiens OX=9606 GN=HNRNPM PE=1 SV=3** |
| 4 | P27694 | 931 | 68723 | 52 (39) | 29 (23) | 3.25 | Replication protein A 70 kDa DNA-binding subunit OS=Homo sapiens OX=9606 GN=RPA1 PE=1 SV=2 |
| 5 | Q96PK6 | 399 | 69620 | 20 (14) | 13 (10) | 0.66 | RNA-binding protein 14 OS=Homo sapiens OX=9606 GN=RBM14 PE=1 SV=2 |
| 6.1 | P16989 | 383 | 40066 | 20 (13) | 15 (10) | 1.21 | Y-box-binding protein 3 OS=Homo sapiens OX=9606 GN=YBX3 PE=1 SV=4 |
| 6.2 | P67809 | 366 | 35903 | 18 (12) | 13 (8) | 1.22 | Nuclease-sensitive element-binding protein 1 OS=Homo sapiens OX=9606 GN=YBX1 PE=1 SV=3 |
| 7 | Q9NRW3 | 313 | 23267 | 15 (10) | 7 (4) | 1.24 | DNA dC->dU-editing enzyme APOBEC-3C OS=Homo sapiens OX=9606 GN=APOBEC3C PE=1 SV=2 |
| 8 | Q9BQE3 | 180 | 50548 | 11 (8) | 8 (8) | 0.66 | Tubulin alpha-1C chain OS=Homo sapiens OX=9606 GN=TUBA1C PE=1 SV=1 |
| 9 | Q02413 | 145 | 114702 | 5 (3) | 4 (3) | 0.09 | Desmoglein-1 OS=Homo sapiens OX=9606 GN=DSG1 PE=1 SV=2 |
| 10 | Q86YZ3 | 123 | 283140 | 13 (4) | 9 (3) | 0.03 | Hornerin OS=Homo sapiens OX=9606 GN=HRNR PE=1 SV=2 |
| 11 | P60709 | 115 | 42052 | 8 (4) | 7 (4) | 0.35 | Actin, cytoplasmic 1 OS=Homo sapiens OX=9606 GN=ACTB PE=1 SV=1 |
| 12 | P07437 | 110 | 50095 | 9 (5) | 7 (4) | 0.38 | Tubulin beta chain OS=Homo sapiens OX=9606 GN=TUBB PE=1 SV=2 |
| 13 | P04406 | 105 | 36201 | 6 (3) | 6 (3) | 0.3 | Glyceraldehyde-3-phosphate dehydrogenase OS=Homo sapiens OX=9606 GN=GAPDH PE=1 SV=3 |
| 14 | P02768 | 103 | 71317 | 10 (7) | 8 (6) | 0.37 | Serum albumin OS=Homo sapiens OX=9606 GN=ALB PE=1 SV=2 |
| 15 | P05109 | 97 | 10885 | 5 (3) | 1 (1) | 0.74 | Protein S100-A8 OS=Homo sapiens OX=9606 GN=S100A8 PE=1 SV=1 |
| 16 | P81605 | 91 | 11391 | 4 (4) | 3 (3) | 1.2 | Dermcidin OS=Homo sapiens OX=9606 GN=DCD PE=1 SV=2 |
| 17 | Q9Y6M1 | 72 | 66195 | 5 (3) | 5 (3) | 0.16 | Insulin-like growth factor 2 mRNA-binding protein 2 OS=Homo sapiens OX=9606 GN=IGF2BP2 PE=1 SV=2 |
| 18 | P15927 | 66 | 29342 | 11 (2) | 7 (2) | 0.24 | Replication protein A 32 kDa subunit OS=Homo sapiens OX=9606 GN=RPA2 PE=1 SV=1 |
| 19 | Q6XE24 | 57 | 48094 | 2 (1) | 2 (1) | 0.07 | RNA-binding motif, single-stranded-interacting protein 3 OS=Homo sapiens OX=9606 GN=RBMS3 PE=1 SV=1 |
| 20 | Q6UWP8 | 53 | 60562 | 2 (1) | 2 (1) | 0.05 | Suprabasin OS=Homo sapiens OX=9606 GN=SBSN PE=1 SV=2 |
| 21 | P11142 | 50 | 71082 | 4 (1) | 4 (1) | 0.05 | Heat shock cognate 71 kDa protein OS=Homo sapiens OX=9606 GN=HSPA8 PE=1 SV=1 |
| 22 | Q14103 | 50 | 38581 | 3 (1) | 3 (1) | 0.09 | Heterogeneous nuclear ribonucleoprotein D0 OS=Homo sapiens OX=9606 GN=HNRNPD PE=1 SV=1 |
| 23 | P12273 | 49 | 16847 | 5 (2) | 5 (2) | 0.44 | Prolactin-inducible protein OS=Homo sapiens OX=9606 GN=PIP PE=1 SV=1 |
| 24 | P30101 | 43 | 57146 | 3 (1) | 3 (1) | 0.06 | Protein disulfide-isomerase A3 OS=Homo sapiens OX=9606 GN=PDIA3 PE=1 SV=4 |
| 25 | O60506 | 41 | 69788 | 2 (1) | 2 (1) | 0.05 | Heterogeneous nuclear ribonucleoprotein Q OS=Homo sapiens OX=9606 GN=SYNCRIP PE=1 SV=2 |
| 26 | P62979 | 39 | 18296 | 2 (1) | 2 (1) | 0.18 | Ubiquitin-40S ribosomal protein S27a OS=Homo sapiens OX=9606 GN=RPS27A PE=1 SV=2 |
| 27 | Q5T749 | 35 | 67172 | 1 (1) | 1 (1) | 0.05 | Keratinocyte proline-rich protein OS=Homo sapiens OX=9606 GN=KPRP PE=1 SV=1 |
| 28 | P32119 | 34 | 22049 | 2 (1) | 2 (1) | 0.15 | Peroxiredoxin-2 OS=Homo sapiens OX=9606 GN=PRDX2 PE=1 SV=5 |
| 29 | Q86V81 | 31 | 26872 | 2 (1) | 2 (1) | 0.12 | THO complex subunit 4 OS=Homo sapiens OX=9606 GN=ALYREF PE=1 SV=3 |
| 30 | Q2PPJ7 | 31 | 213088 | 9 (1) | 6 (1) | 0.02 | Ral GTPase-activating protein subunit alpha-2 OS=Homo sapiens OX=9606 GN=RALGAPA2 PE=1 SV=2 |
| 31 | P25311 | 31 | 34465 | 4 (1) | 4 (1) | 0.1 | Zinc-alpha-2-glycoprotein OS=Homo sapiens OX=9606 GN=AZGP1 PE=1 SV=2 |
| 32 | P0C0S5 | 31 | 13545 | 3 (2) | 3 (2) | 0.57 | Histone H2A.Z OS=Homo sapiens OX=9606 GN=H2AFZ PE=1 SV=2 |
| 33 | P25705 | 31 | 59828 | 6 (1) | 5 (1) | 0.05 | ATP synthase subunit alpha, mitochondrial OS=Homo sapiens OX=9606 GN=ATP5F1A PE=1 SV=1 |
| 34 | P12956 | 29 | 70084 | 3 (1) | 2 (1) | 0.05 | X-ray repair cross-complementing protein 6 OS=Homo sapiens OX=9606 GN=XRCC6 PE=1 SV=2 |
| 35 | P09038 | 29 | 31093 | 2 (1) | 2 (1) | 0.11 | Fibroblast growth factor 2 OS=Homo sapiens OX=9606 GN=FGF2 PE=1 SV=3 |
| 36 | Q8IWZ3 | 28 | 271286 | 2 (1) | 2 (1) | 0.01 | Ankyrin repeat and KH domain-containing protein 1 OS=Homo sapiens OX=9606 GN=ANKHD1 PE=1 SV=1 |
| 37 | A6NI56 | 27 | 76422 | 2 (1) | 2 (1) | 0.04 | Coiled-coil domain-containing protein 154 OS=Homo sapiens OX=9606 GN=CCDC154 PE=2 SV=4 |
| 38 | Q6NXT2 | 27 | 15318 | 3 (1) | 3 (1) | 0.22 | Histone H3.3C OS=Homo sapiens OX=9606 GN=H3F3C PE=1 SV=3 |
| 39 | Q4AC94 | 27 | 262621 | 2 (1) | 2 (1) | 0.01 | C2 domain-containing protein 3 OS=Homo sapiens OX=9606 GN=C2CD3 PE=1 SV=4 |
| 40 | Q15149 | 27 | 533462 | 17 (1) | 14 (1) | 0.01 | Plectin OS=Homo sapiens OX=9606 GN=PLEC PE=1 SV=3 |
| 41 | O00555 | 26 | 283782 | 8 (1) | 6 (1) | 0.01 | Voltage-dependent P/Q-type calcium channel subunit alpha-1A OS=Homo sapiens OX=9606 GN=CACNA1A PE=1 SV=2 |
| 42 | Q01968 | 26 | 105392 | 6 (1) | 1 (1) | 0.03 | Inositol polyphosphate 5-phosphatase OCRL-1 OS=Homo sapiens OX=9606 GN=OCRL PE=1 SV=3 |
| 43 | Q96DN5 | 26 | 125137 | 3 (1) | 3 (1) | 0.03 | TBC1 domain family member 31 OS=Homo sapiens OX=9606 GN=TBC1D31 PE=1 SV=2 |
| 44 | Q99698 | 25 | 434169 | 7 (1) | 6 (1) | 0.01 | Lysosomal-trafficking regulator OS=Homo sapiens OX=9606 GN=LYST PE=1 SV=3 |
| 45 | P14618 | 25 | 58470 | 4 (1) | 4 (1) | 0.06 | Pyruvate kinase PKM OS=Homo sapiens OX=9606 GN=PKM PE=1 SV=4 |
| 46 | Q8N3T1 | 25 | 74043 | 1 (1) | 1 (1) | 0.04 | Polypeptide N-acetylgalactosaminyltransferase 15 OS=Homo sapiens OX=9606 GN=GALNT15 PE=2 SV=2 |
| 47 | P02042 | 25 | 16159 | 1 (1) | 1 (1) | 0.21 | Hemoglobin subunit delta OS=Homo sapiens OX=9606 GN=HBD PE=1 SV=2 |
| 48 | Q15645 | 25 | 48863 | 2 (1) | 2 (1) | 0.07 | Pachytene checkpoint protein 2 homolog OS=Homo sapiens OX=9606 GN=TRIP13 PE=1 SV=2 |
| 49 | Q9P2P6 | 24 | 521840 | 74 (1) | 6 (1) | 0.01 | StAR-related lipid transfer protein 9 OS=Homo sapiens OX=9606 GN=STARD9 PE=1 SV=3 |
| 50 | Q13535 | 24 | 304764 | 8 (1) | 4 (1) | 0.01 | Serine/threonine-protein kinase ATR OS=Homo sapiens OX=9606 GN=ATR PE=1 SV=3 |
| 51 | Q9ULK5 | 24 | 59905 | 2 (1) | 1 (1) | 0.05 | Vang-like protein 2 OS=Homo sapiens OX=9606 GN=VANGL2 PE=1 SV=2 |
| 52 | Q5TCY1 | 23 | 142992 | 9 (1) | 5 (1) | 0.02 | Tau-tubulin kinase 1 OS=Homo sapiens OX=9606 GN=TTBK1 PE=1 SV=2 |
| 53 | P54727 | 23 | 43202 | 2 (1) | 2 (1) | 0.08 | UV excision repair protein RAD23 homolog B OS=Homo sapiens OX=9606 GN=RAD23B PE=1 SV=1 |
| 54 | Q8N1K5 | 23 | 74033 | 2 (1) | 2 (1) | 0.04 | Protein THEMIS OS=Homo sapiens OX=9606 GN=THEMIS PE=1 SV=3 |
| 55 | A6NKL6 | 23 | 64345 | 3 (1) | 2 (1) | 0.05 | Transmembrane protein 200C OS=Homo sapiens OX=9606 GN=TMEM200C PE=2 SV=2 |
| 56 | Q04917 | 22 | 28372 | 2 (1) | 2 (1) | 0.12 | 14-3-3 protein eta OS=Homo sapiens OX=9606 GN=YWHAH PE=1 SV=4 |
| 57 | Q5T5U3 | 22 | 218567 | 4 (1) | 4 (1) | 0.01 | Rho GTPase-activating protein 21 OS=Homo sapiens OX=9606 GN=ARHGAP21 PE=1 SV=1 |
| 58 | Q9NR82 | 22 | 103141 | 4 (1) | 4 (1) | 0.03 | Potassium voltage-gated channel subfamily KQT member 5 OS=Homo sapiens OX=9606 GN=KCNQ5 PE=1 SV=3 |
| 59 | Q9H4I2 | 21 | 105447 | 2 (1) | 2 (1) | 0.03 | Zinc fingers and homeoboxes protein 3 OS=Homo sapiens OX=9606 GN=ZHX3 PE=1 SV=3 |
| 60 | Q99250 | 20 | 229994 | 6 (1) | 3 (1) | 0.01 | Sodium channel protein type 2 subunit alpha OS=Homo sapiens OX=9606 GN=SCN2A PE=1 SV=3 |
| 61 | P38159 | 18 | 42306 | 4 (1) | 4 (1) | 0.08 | RNA-binding motif protein, X chromosome OS=Homo sapiens OX=9606 GN=RBMX PE=1 SV=3 |
| 62 | Q9P2F5 | 18 | 103748 | 1 (1) | 1 (1) | 0.03 | Storkhead-box protein 2 OS=Homo sapiens OX=9606 GN=STOX2 PE=2 SV=2 |
| 63 | Q9Y4L1 | 18 | 111494 | 23 (1) | 2 (1) | 0.03 | Hypoxia up-regulated protein 1 OS=Homo sapiens OX=9606 GN=HYOU1 PE=1 SV=1 |
| 64 | Q5T699 | 18 | 47097 | 1 (1) | 1 (1) | 0.07 | Putative uncharacterized protein C6orf183 OS=Homo sapiens OX=9606 GN=C6orf183 PE=5 SV=3 |
| 65 | Q02928 | 17 | 59766 | 3 (1) | 2 (1) | 0.06 | Cytochrome P450 4A11 OS=Homo sapiens OX=9606 GN=CYP4A11 PE=1 SV=1 |
| 66 | Q2M3R5 | 15 | 40380 | 1 (1) | 1 (1) | 0.08 | Solute carrier family 35 member G1 OS=Homo sapiens OX=9606 GN=SLC35G1 PE=1 SV=1 |
| 67 | Q14765 | 15 | 86399 | 2 (2) | 1 (1) | 0.04 | Signal transducer and activator of transcription 4 OS=Homo sapiens OX=9606 GN=STAT4 PE=1 SV=1 |
| 68 | P16219 | 14 | 44611 | 2 (1) | 2 (1) | 0.07 | Short-chain specific acyl-CoA dehydrogenase, mitochondrial OS=Homo sapiens OX=9606 GN=ACADS PE=1 SV=1 |
